# Nerve Growth Factor Biodelivery: A Limiting Step in Moving Toward Extensive Clinical Application?

**DOI:** 10.3389/fnins.2021.695592

**Published:** 2021-07-15

**Authors:** Giuseppe Alastra, Luigi Aloe, Vito Antonio Baldassarro, Laura Calzà, Maura Cescatti, Jason Thomas Duskey, Maria Letizia Focarete, Daria Giacomini, Luciana Giardino, Valentina Giraldi, Luca Lorenzini, Marzia Moretti, Irene Parmeggiani, Michele Sannia, Giovanni Tosi

**Affiliations:** ^1^Interdepartmental Centre for Industrial Research in Health Sciences and Technologies, University of Bologna, Bologna, Italy; ^2^IRET Foundation, Bologna, Italy; ^3^Department of Pharmacy and Biotechnology, University of Bologna, Bologna, Italy; ^4^Nanotech Laboratory, TeFarTI Center, Department of Life Sciences, University of Modena and Reggio Emilia, Modena, Italy; ^5^Department of Chemistry “Giacomo Ciamician”, University of Bologna, Bologna, Italy; ^6^Department of Veterinary Medical Sciences, University of Bologna, Bologna, Italy

**Keywords:** nerve growth factor, nanomedicine, drug delivery, electrospinning, hydrogels

## Abstract

Nerve growth factor (NGF) was the first-discovered member of the neurotrophin family, a class of bioactive molecules which exerts powerful biological effects on the CNS and other peripheral tissues, not only during development, but also during adulthood. While these molecules have long been regarded as potential drugs to combat acute and chronic neurodegenerative processes, as evidenced by the extensive data on their neuroprotective properties, their clinical application has been hindered by their unexpected side effects, as well as by difficulties in defining appropriate dosing and administration strategies. This paper reviews aspects related to the endogenous production of NGF in healthy and pathological conditions, along with conventional and biomaterial-assisted delivery strategies, in an attempt to clarify the impediments to the clinical application of this powerful molecule.

## Introduction

Since its discovery in the 1950s (Levi-Montalcini, 1987) and the award of the Nobel Prize to Rita Levi-Montalcini and Stanley Cohen for their discoveries of growth factors in 1986, the number of basic science discoveries and preclinical studies supporting the use of nerve growth factor (NGF) for therapeutic purposes has constantly increased over the years, principally for neurodegenerative diseases (Alzheimer’s disease, AD; and Parkinson’s disease, PD) and brain injuries (perinatal hypoxia/ischemia, traumatic brain, and spinal cord injury), but also retinopathies, optic nerve degeneration, and peripheral neuropathies associated with diabetes and HIV. The potential applications have also extended from the nervous system as the primary target to an increasing number of tissues and organs, including epithelial tissue, parenchymal organs, and the osteoarticular system (Manni et al., 2013; Rocco et al., 2018), as well as inflammation (Skaper, 2017) and cancer (Griffin et al., 2018).

The road toward the clinical translation of NGF, however, has encountered numerous obstacles. In spite of the success of Genentech in translating NGF production from male mouse salivary gland extract (Bocchini and Angeletti, 1969) to the recombinant technology of the human form (Altar et al., 1991; Rogers, 1996), results from early clinical trials led Genentech to terminate the rhNGF (recombinant human NGF) project. This was based on side effects observed in two sets of phase II clinical trials, suggesting that despite the efficacy of rhNGF administration at ameliorating the symptoms associated with both diabetic polyneuropathy and HIV-related neuropathy, side effects were dose limiting for NGF (Apfel, 2002). Moreover, a large-scale phase III clinical trial of 1019 patients randomized to receive either rhNGF or placebo for 48 weeks failed to confirm these earlier indications of efficacy (Apfel, 2002). Intravenous (IV), subcutaneous (SC), and intradermal (ID) NGF injection induces myalgia, mechanic and thermal hyperalgesia, emerging rapidly after injection and lasting for weeks (Mizumura and Murase, 2015). As concerns human studies, SC or intracerebroventricular (ICV) administration of rhNGF to healthy subjects or patients with diabetic polyneuropathy, HIV-associated peripheral neuropathy (SC, 0.03–1 μg/kg), AD (ICV, 75 μg/day for 3 months), PD (ICV, 3.3 mg infused over 23 days) or hypoxic-ischemic perinatal brain injury (0.1 mg/day for 10 days) (reviewed by Tria et al., 1994; Mizumura and Murase, 2015) always produced hyperalgesia at the injection site, and in some cases also mild to moderate-severe transient muscle pain (Petty et al., 1994; Rogers, 1996) (clinical trial NCT00000842).

Actually, the current evidence regarding the painful side effects of NGF administration is taking pharmacological research in two new directions: development of humanized anti-NGF monoclonal antibodies (anti-NGF mAbs) for conditions as osteoarthritis, lower back pain, and interstitial cystitis (Wise et al., 2021), and synthesis of TrkA ligands in an attempt to overcome this severe and limiting side effect (Carleton et al., 2018; Bagal et al., 2019). The rhNGF has finally received FDA approval as Cenegermin^®^ eye drops by Dompé, first-in-class with the potential to completely heal rare neurotrophic keratitis (clinical trials NCT04293549, NCT03836859, NCT02101281, NCT03019627).

However, the major obstacles to clinical translation of NGF are also due to other factors, such as the biodistribution of this large molecule, including its crossing of blood-tissue barriers, and to issues of dosage, since NGF is produced by many different cell types (Gostynska et al., 2020), and because endogenous NGF production is altered in many of the pathologies included in a tentative list of potential targets for the NGF drug.

This review addresses some of these major issues affecting the development of innovative NGF delivery solutions, discussing possible reasons for their success, and in many cases their failures. Sections “Parenteral Administration” and “Topical Application” refer to both preclinical and clinical studies, these latter also indicated by the respective clinicaltrials.gov code; section “Biomaterial-Assisted Delivery” refers to *in vitro* and preclinical studies.

## Parenteral Administration

### NGF Endogenous Levels, Biodistribution and Metabolism

In the body, NGF is produced according to a delicate balance that varies from tissue to tissue also according to specific diseases and pathological states (Lorenzini et al., 2021), and that can be reflected by NGF blood levels

There is little data available on the biodistribution of exogenously administered NGF. In the initial human applications and clinical trials, mouse NGF or recombinant human NGF (rhNGF) was SC (Petty et al., 1994; Rogers, 1996; Apfel et al., 1998, 2000; McArthur et al., 2000; Schifitto et al., 2001) (clinical trial NCT00000842) or ICV administered (Olson et al., 1992; Jönhagen et al., 1998; Chiaretti et al., 2005, 2008), but basic information on absorption, distribution and excretion following parenteral administration derives from animal studies, in particular in adult rats and in cynomolgus monkeys. In this few biodistribution studies, mouse NGF was administrated intravenously (IV) and SC in rats as a single injection (35 μg/kg, single dose) or by continuous infusion via osmotic mini-pump (50–450 μg/pump) (Tria et al., 1994). In monkeys, rhNGF was administered SC (2 mg/kg), and pharmacokinetic analysis was conducted after single and multiple doses (for 15 days, every other day) (Nguyen et al., 2000). In both studies, the maximum plasma concentrations (*C*_*max*_) confirmed that the drug is absorbed after SC administration. In rats, the maximum blood concentration of NGF after SC administration was 65-fold lower than after IV injection. The calculated time to reach maximum plasma concentrations (*T*_*max*_) are very similar in two studies, despite the different doses employed. Multiple dosing in monkeys shifts the *T*_*max*_ from a mean value of 2.5 to 3.3 h (Nguyen et al., 2000; Tria et al., 1994).

Subcutaneous administration via osmotic mini-pump (450 μg/pump, corresponding to 37.5 μg/day) in rats resulted in detectable NGF plasma levels after 6 h, reaching peak values during day 1, confirming the *T*_*max*_ delay after multiple dosing (Tria et al., 1994). IV injection allows calculation of the half-life distribution phase (*t*_1__/__2__α_), reached in 5–6 min, indicating a rapid disappearance from plasma, probably due to the binding of NGF to the α2-macroglobulin cleared from blood by hepatocytes (Tria et al., 1994). With regard to NGF metabolism, no degradation products were observed in plasma after immunoprecipitation and SDS-PAGE, suggesting a long-term stability of the protein (Tria et al., 1994; Nguyen et al., 2000). Table 1 summarizes the basic pharmacokinetic parameters following comparable administration routes.

**TABLE 1:** Main PK parameters evaluated in animal studies, following comparable single subcutaneous injection (data from Tria et al., 1994; Nguyen et al., 2000).

	Monkeys	Rats
	
	rhNGF	mNGF
Dose	2 mg/kg	35 μg/kg
*C*_*max*_ (ng/ml)	1300 ± 120	3.57 ± 0.33
*T*_*max*_ (h)	2.5 ± 2.7	3.20 ± 0.49
*t*_1/2β_ (h)	4.1 ± 1.0	4.47 ± 0.15

*C_max_, maximum plasma concentration; *T*_*max*_, time to reach maximum plasma concentration; *t*_1/2β_, elimination phase half-life.*

Data regarding tissue distribution were obtained following the administration of radiolabeled rhNGF (^125^I-rhNGF) in primates (multiple dosing, for 15 days, every other day). The large central volume of distribution (V_2_/F, 827 ml/kg) indicated distribution in extravascular tissues. The organs were then collected at 8 and 24 h following dose 1 and dose 15. In non-neuronal tissues ^125^I-rhNGF was detected at both time points and doses in all studied tissues, particularly in the thyroid, adrenals, kidneys, liver, spleen, peripheral and axillary tissues, and at the injection site. As expected, the radiolabeled drug was observed in the peripheral nervous system, whereas it was minimal in the spinal cord, and absent in the brain (Nguyen et al., 2000).

The plasma elimination half-life (*t*_1/2β_) is very close both in rats and in monkeys (Table 1) and can be extended by varying the administration route, e.g., 4.47 h in SC versus 2.30 h in IV injection in rats. The administration schedule does not appear to affect this parameter, e.g., 4.1 h versus 4.8 h following single and multiple dosing. The clearance values (Cl) are not affected by administration route, e.g., 6.38 ml/min/kg in SC versus 6.93 ml/min/kg in IV injection in rats, but decrease following multiple administration, e.g., 140 ml/kg/hr versus 63 ml/kg/hr following single and multiple (Tria et al., 1994; Nguyen et al., 2000).

With regard to NGF metabolism, analysis of radiolabeled NGF in monkeys shows that urinary excretion represents the main route of elimination, although traces of radioactivity were found in the feces. No difference in elimination pattern was observed between 24 and 120 h following single and multiple dosing (Tria et al., 1994; Nguyen et al., 2000). There is also very little data available on specific NGF metabolic products, derived from immunoprecipitation and SDS-PAGE in monkey tissue lysates. In non-neuronal tissues, low molecular mass bands were observed in the kidney, liver and spleen, indicating that intensive metabolism occurs in these organs. SDS-PAGE from lysate of sympathetic ganglia and dorsal root ganglia also show intense NGF metabolism, while material present in the peripheral nerves (radial, sciatic, and tibial) was mostly negative (Tria et al., 1994; Nguyen et al., 2000).

### NGF and the Blood Brain Barrier

Peripheral administration of NGF to target the CNS is limited by the poor ability of this molecule to cross the blood-brain barrier (BBB), and by peripheral enzymatic degradation. The unique properties of the BBB stem from CNS capillary histology, where endothelial cells are held together by tight junctions which limit the paracellular flux of solutes, and the presence of specific transporters which regulate the passage of molecules to the CNSs. Endothelial cells are covered by mural cells (pericytes and smooth muscle cells) which contribute to the dynamics of BBB control, and the microvascular tube is also surrounded by the inner vascular and the outer parenchymal basement membrane, providing an anchor for many signaling processes and an additional barrier for cells and molecules accessing the CNS. The blood vessels also interact with different immune cells, mainly perivascular macrophages, and microglial cells, representing the first line of innate immunity. Lastly, the direct bridge from the microvessel to the neurons is the astrocyte, a glial cell with extending processes which completely envelop the vascular tube, connecting the microvessels to the neurons (Daneman and Prat, 2015).

While the restrictive nature of the BBB allows for proper neuronal function and protection of the neural tissue, and maintains CNS homeostasis, it also constitutes an obstacle for drug delivery. Whether NGF can penetrate the BBB and be absorbed by the brain tissue, and under what conditions, is still controversial, but the poor permeability of NGF through the BBB under physiological conditions has been widely described (Pan et al., 1998). However, the BBB is not a fixed structure, undergoing pathophysiological adaptations which are not yet fully understood. Although BBB formation starts during the embryonic stage, soon after vessel formation in the developing CNS (E11, in rats), the system continues to mature following birth, increasing the strength of the paracellular barrier and expression of the efflux transporter (Blanchette and Daneman, 2015), while pathological conditions, particularly inflammation, are known to modify its structure and dynamics. In general, different diseases (e.g., stroke, multiple sclerosis, epilepsy) are characterized by the internalization and down-regulation of tight junctions, increased rates of transcytosis, increased expression of adhesion molecules for leukocytes leading to increased leukocyte extravasation, degradation of the basal membrane, and reduced microvessel coverage by pericytes and astrocytes (Profaci et al., 2020). Specific pathologies may therefore offer time window opportunities for exploiting altered BBB permeability and increasing NGF transportation to the CNS.

Strategies to overcome the BBB for the CNS delivery of large molecules as NGF represent a major goal, driving alternative routes of administration [see section “Intranasal (IN)” and “Eye Drops”] and pharmaceutical technologies as nanocarrier (see section “Nanomedicines for NGF Delivery”).

## Topical Application

### Intranasal (IN)

Due to its large surface area, the high degree of vascularization, and the “nose-to-brain” pathways, the nasal cavity is an interesting portal for systemic delivery and to by-pass the BBB. The advantages of nose-to-brain drug delivery include safety and avoidance of the hepatic first pass metabolism, as well as its non-invasive nature and high patient compliance (Colombo et al., 2011). Limitations include the possible dosing volume through the nasal cavity and the consequent total amount of drug delivered systemically or into the brain (Dong, 2018), active mucociliary clearance of the mucosa, short retention time for drug absorption, low permeability for hydrophilic drugs, and low central nervous system (CNS) delivery for proteins (Erdõ et al., 2018). For these reasons, many strategies are currently being tested to enhance drug transport and distribution through the “nose-to-brain” pathways.

**FIGURE 1:**
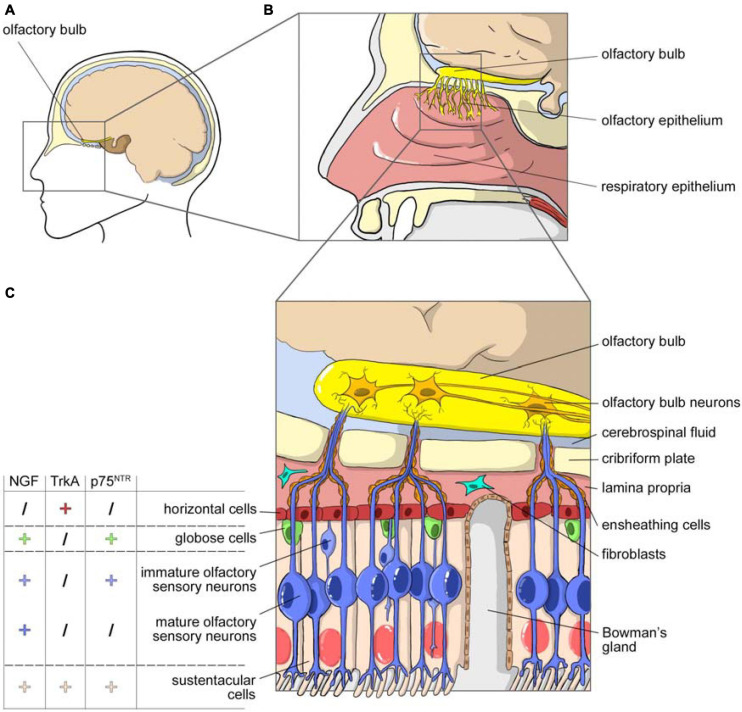
NGF and olfactory system. **(A)** Sagittal view of the head with highlighted the area occupied by the nasal cavity. **(B)** Magnification of the nasal cavity with, in the square, the area occupied by the olfactory epithelium. **(C)** Cell composition of the olfactory part of the nasal cavity, mucosa, olfactory epithelium, and lamina propria. The olfactory epithelium is surrounded by layer globose and horizontal basal cells. It consists in many cellular types: sustentacular cells, Bowman’s glands producing mucus, immature olfactory neurons and mature olfactory sensory neurons that project their axons toward the olfactory bulb through the cribriform plate. Axons are enclosed by olfactory ensheating cells and olfactory nerve fibroblasts. The picture includes data on the expression of NGF, TrkA and p75^NTR^ in the different cellular populations of the olfactory epithelium (from Feron et al., 2008).

To give a brief anatomical overview, the nasal cavity consists of three anatomical areas, the nasal vestibule, respiratory region, and olfactory region, each characterized by different mucosal epithelia. Figure 1 shows the cell composition of the olfactory part of nasal cavity, mucosa, olfactory epithelium and lamina propria, including the detail of the NGF, TrkA, and p75^*NTR*^ expression on different cell types.

The respiratory epithelium is a ciliated pseudostratified columnar epithelium composed by four main cell types – ciliated and non-ciliated columnar cells, basal or horizontal cells and goblet cells – with high vascularization, supplied by the arterial branch of the maxillary artery. A mucus gel layer covers the epithelium, that, together with ciliary tip movements, constitutes the first protective barrier against inhaled particulates and irritants. The respiratory mucus layer is renewed every 10–20 min (Pardeshi and Belgamwar, 2013).

While the respiratory region is mainly involved in systemic drug absorption, the olfactory area is important not only for the ability of its neurons to provide the sense of smell, but also for the “nose-to-brain path,” which delivers drugs directly into the brain. The olfactory mucosa consists of a ciliated chemosensory pseudostratified columnar epithelium that contains three types of cells – olfactory sensory neurons, immature olfactory sensory neurons and supporting (or sustentacular) cells – all connected by tight junctions (Figures 1A,B). The cilia are non-motile, and the overlying mucus gel has a very slow turnover (several days).

The olfactory mucosa presents the main inter-species anatomical differences, an aspect which must be considered when translating animal data to humans. In humans, this mucosa covers 10% of the total surface area, while in rodents, the most widely used species for intranasal administration studies, it can constitute up to 50% of the total area. This is an important aspect, because results obtained from animal models do not always correlate with those of humans, a discrepancy which probably comes from an insufficient consideration of the anatomical and physiological differences between the respective nasal cavities (Cho et al., 2010). Rodents are more widely used for preliminary nose-to-brain drug absorption studies, while rabbits and dogs are used for pharmacokinetic studies.

Drugs transferred from the olfactory mucosa to the CNS bypassing the BBB follow two pathways, olfactory and trigeminal, with molecular transfer taking place outside or within the nerve axon. The olfactory path includes the neuronal cells of the olfactory epithelium, and the lamina propria and the olfactory bulb in the CNS. The olfactory bulb then projects to the cortex, amygdala and hypothalamus, providing an anatomical link between nasal administration and the brain structures (Khan et al., 2017). The trigeminal path consists of the trigeminal nerve with its three major branches, ophthalmic, maxillary, and mandibular, thus promoting the entrance of drugs to the caudal and rostral parts of the brain. The olfactory path delivers drugs to the rostral areas of the brain only, whereas the trigeminal pathway delivers to both the rostral and caudal areas.

Following drug administration into the nasal cavity, the first step of absorption is the passage through the mucus layer and ciliary movement. After crossing this barrier, several mechanisms are involved in the transmucosal transfer, such as the paracellular pathway (the passive transport of molecules between cells), or the transcellular pathway (active transport of the drug across the cells). Carrier-mediated transport, transcytosis, and transport through the intercellular tight junctions are other possible pathways.

The entry of a wide range of molecules into olfactory sensory neurons via an intracellular mechanism such as pinocytosis or receptor-mediated endocytosis was first demonstrated for BDNF (Deckner et al., 1993), and more recently for other drugs such as ribavirin, an antiviral drug potentially useful for the treatment of viral infections in both humans and animals (Colombo et al., 2011; Giuliani et al., 2018). This is the mechanism used by many viruses such as poliovirus or herpesvirus, as well as by the latest example, the SARS-CoV-2 virus. Following internalization in olfactory neurons, the molecules (or viruses) run down the soma via retrograde axonal transport. Neuronal transport is considered a slow process. For example, intranasal delivery of 70 μg radiolabeled BDNF, CNTF, NT-4, or erythropoietin (EPO) resulted in 0.1–1.0 nM neurotrophin concentrations within 25 min in brain parenchyma (Alcalá-Barraza et al., 2010). Intranasal studies using labeled IGF-1 suggest that the rapid distribution toward the CNS (∼30 min) is due to extracellular convection or intracellular transport rather than to diffusion (Thorne et al., 2004). Other studies report 45 min for the axonal transport phase (Crowe et al., 2018).

Despite the presence of tight junctions (TJs), the use of intercellular spaces has been hypothesized. These spaces are generated by channels through which proteins, peptides (such as insulin, IGF-1, albumin) and even stem cells can reach the CNS, as demonstrated in the nasal mucosa, and by a transient loosening of the BBB by decreasing expression of TJ proteins such as claudin-1, occludin, and tricellulin (Jackson et al., 2017).

Although interest in this delivery route for preclinical and clinical studies is increasing, very few studies of NGF pharmacokinetics or biodistribution are available. In a study on the Sprague Dawley rat hippocampus, for example, the bioavailability of intranasally administered NGF with or without chitosan was ∼14 fold greater than the group treated with NGF without chitosan (Vaka et al., 2009). In a preclinical model of AD, polymeric nanoparticles appear to be promising carriers for the nose-to-brain delivery of drugs (Rabiee et al., 2021). Following IN administration, rhNGF reached the brain within an hour, achieving a concentration of 3400 pM in the olfactory bulb, 660–2200 pM in other brain regions and, 240 and 180 pM in the hippocampus and the amygdala, respectively, while, little or no rhNGF was found in the brain following IV administration (Chen et al., 1998). The therapeutic efficacy of IN NGF administration has also been evaluated in many other brain diseases, and in clinical trials of traumatic brain injury, acute ischemic stroke and frontotemporal dementia (Eftimiadi et al., 2021). Notably, no systemic or local side-effects have been described in clinical trials using IN NGF administration in both adult (10 μl of NGF at 200 μg/ml concentration, daily, for a 1-year period) (de Bellis et al., 2018) and pediatric patients (0.1 mg/kg, three times daily for 7 consecutive days) (Chiaretti et al., 2020).

### Eye Drops

The eye is regarded as one of the main therapeutic targets for NGF topical treatments. Local application of NGF exerts a healing action on corneal and cutaneous ulcers associated with pathological conditions such as inflammation, diabetes and rheumatoid arthritis (Aloe et al., 2008), and the use of NGF as a drug in ophthalmology is the best characterized and developed clinical use of this neurotrophin (Eftimiadi et al., 2021). Since the initial discovery that goldfish retinal cells are receptive to NGF action (Turner and Delaney, 1979), many studies have shown the potential therapeutic use of NGF to treat ophthalmic diseases (Aloe et al., 2012), leading to a number of pre-clinical research studies and clinical trials on different eye-related pathologies (Aloe et al., 2012; Manni et al., 2013).

The most recent research into NGF treatments has focused on neurotrophic keratitis, dry eye disease, optic neuropathy and optic pathway glioma (Eftimiadi et al., 2021), and the treatment of corneal ulcers of different etiologies, treated by topical NGF application in more than 200 patients, is of major interest (Lambiase et al., 2012). However, it was only in 2018, following 30 years of clinical trials (Bonini et al., 2018), that research finally led to the approval of a rhNGF produced in bacteria, named Cenegermin (Oxervate^TM^; Dompè Farmaceutici SpA, Milan, Italy) for the treatment of neurotrophic keratitis. The topical administration of NGF leads to complete corneal healing (Bonini et al., 2018; Deeks and Lamb, 2020; Pflugfelder et al., 2020), without inducing the development of pain and circulating anti-NGF antibodies (Lambiase et al., 2007a).

But the retinal cells in the eye are part of the CNS and constitutes the visual system together with the brain areas receiving retinal input. The retina, which is part of the posterior segment, is composed of different layers of nerve cell bodies organized in nuclear and synaptic layers, transforming light into nerve signals. From the retina, the retinal ganglion cell (RGCs) axons form the nerve fibers which converge in the optic disk and form the optic nerve.

**FIGURE 2:**
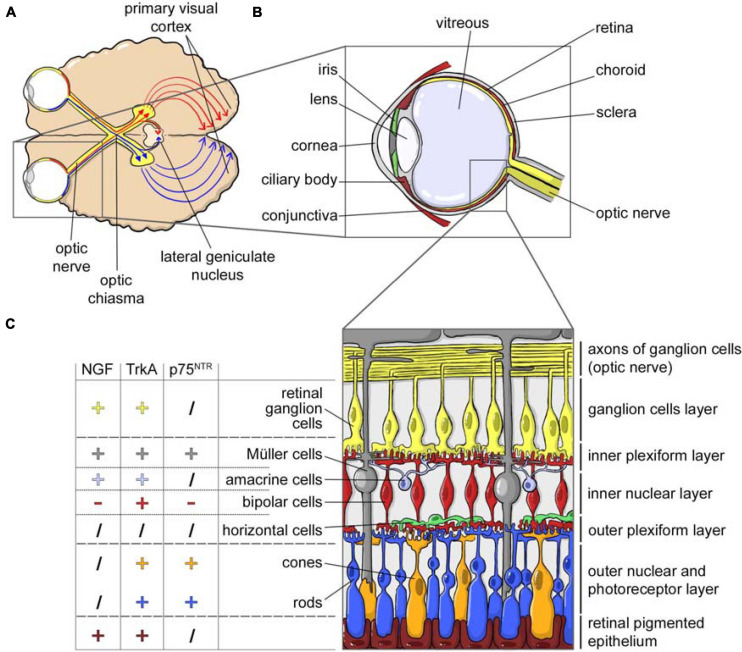
NGF and the visual system. **(A)** Horizontal cross section of the brain, showing the optic nerves originating from retina and crossing at the optic chiasm. Each optic tract travels to its corresponding cerebral hemisphere to reach the lateral geniculate nucleus in the thalamus and to the contralateral hemisphere to reach the primary visual cortex. **(B)** Horizontal cross section of the eye showing the anterior (cornea, conjunctiva, iris, ciliary body, and lens) and a posterior (sclera, choroid, retina, and optic nerve) ocular segment, filled with the vitreous fluid. From the retina, the retinal ganglion cells axons form the nerve fibers converging in the optic disk and forming the optic nerve. **(C)** NGF, TrkA, and p75^NTR^ expression in the different cellular populations of the retina (data from Garcia et al., 2017).

Thanks to this neural connection to the brain, the topical application of NGF on eye is also regarded as a delivery route to the brain. In fact, and in addition to innervating primary visual areas, RGCs also extend their projections to the hypothalamus and direct/indirect projections to different limbic structures including the hippocampus and the septum (Tirassa et al., 2018; Murcia-Belmonte and Erskine, 2019; Eftimiadi et al., 2021). Figure 2 shows the structures of the visual system and in particular of the eye (Figures 2A,B), including the detail of the cell composition of the retina and the expression of NGF, TrkA and p75^*NTR*^ in the different cell populations (Figure 2C).

Although the eye has a number of anatomical and physiological barriers which limit the absorption and transport of molecules, topical application is nevertheless highly appealing. Although bioavailability and efficacy after this route are lower than a number of injection routes in different eye compartments (intravitreal, subconjunctival, and retrobulbar), different drugs are still capable of reaching the posterior segment of the eye. Topical application also reduces the chance of systemic side effects, and the drug can even be self-administered as eye drops.

The administration of NGF to the target areas of the brain via the ophthalmic route is theoretically hindered by the molecular weight of the active form of NGF (14.5 KDa) which does not permit its passage through the cornea. However, NGF is unexpectedly absorbed, albeit at a low concentration, reaching the retina, optic nerve and finally the brain (Lambiase et al., 2005; Di Fausto et al., 2007; Lambiase et al., 2007b) via different paths. From the optical surface, several routes direct the transport of the molecule to the posterior segment (Maurice, 2002; Koevary, 2005). NGF receptors are highly expressed throughout the visual system (Wang et al., 2014), and its voyage starts by binding the high affinity receptor TrkA in the anterior part of the eye (Roberti et al., 2014).

Nerve growth factor appears to be transported mainly by the *trans*-conjunctive/*trans*-sclera pathways, although systemic absorption and passage through the retrobulbar space have also been hypothesized (Maurice, 2002; Koevary et al., 2003).

Following the passage from the anterior to the posterior part of the eye, the cells in the retina and the RGCs transport NGF along their axons via anterograde or retrograde mechanisms (Carmignoto et al., 1991), indeed anterograde transport and systemic absorption may explain the increased levels of the molecule in the contralateral eye. NGF eye drops also induce c-Fos in the neurons of the primary visual areas of the CNS, supraoptic and paraventricular nuclei, hippocampus, frontal cortex and amygdala, indicating that all the retinal pathways are activated and that NGF also acts through post-synaptic modulation of cells localized in different brain areas which receive the retinal signals, either directly or indirectly (Tirassa et al., 2018).

An animal study on rats using radiolabeled NGF demonstrated that following eye drop administration, the molecule is present in the conjunctiva, sclera, choroid, retina and optic nerve. In the retina and the optic nerve, NGF was detected as early as 2 h after administration, reaching maximum level at 6 h and disappearing from the eye tissues after 48 h (Lambiase et al., 2005).

Ocular and intranasal application, with their ease of delivery, offer attractive alternatives to the systemic delivery of NGF, bypassing the BBB (Frey et al., 1997; Thorne and Frey, 2001; Aloe et al., 2014). A drawback is the low delivery efficiency. Moreover, the specificity of the treatment is uncertain and highly variable, with unpredictable, albeit minimal systemic effects.

### Skin

Topical NGF applications also include the skin, where it acts locally and is highly effective in wound healing promotion. The cellular actors involved in epithelial tissue repair (keratinocytes, dermal fibroblasts, and myofibroblasts) are cells which produce or respond to NGF, expressing the TrkA high-affinity receptor (Palazzo et al., 2012; Matsumura et al., 2015; Samarasena et al., 2015). In this context, NGF also exerts an angiogenic action on endothelial cells (Calzà et al., 2001; Nico et al., 2008), a direct action on inflammatory and immune cells (Minnone et al., 2017), and a direct effect on the thinly myelinated Aδ- or unmyelinated C-fibers that innervate the dermis and epidermis (Indo, 2010). This is also demonstrated by the role of endogenous NGF in skin and mucosal wound healing in various animal models and human pathologies (Levi-Montalcini, 1987; Chéret et al., 2013).

Taking this evidence as a starting point, several reports have described the positive effect of NGF in epithelial wound healing, including chronic non-healing cutaneous ulcers in diabetic rodent models, where a defect of endogenous NGF is supposed (Tiaka et al., 2011). Our group demonstrated *in vitro* that NGF action is directed at the main cell types involved in wound healing (keratinocytes, fibroblasts, and endothelial cells), as well as at hyperglycemic conditions which mimic the pathological microenvironment of diabetes (Gostynska et al., 2019). We also tested the efficacy of a non-algogenic NGF derivative (hNGFP61S/R100E), named CHF6467 (Chiesi Farmaceutici). This molecule is a rhNGF containing an amino acid substitution, which removed the NGF-related hyperalgesic effect, while maintaining its ability to induce wound healing. CHF6467 treatments of pressure ulcers in diabetic mice accelerated skin repair, increasing re-epithelization, re-innervation, and re-vascularization (Giuliani et al., 2020). Our results confirmed other studies (Muangman et al., 2004), with the remarkable difference that we used a non-algogenic rhNGF, thus potentially overcoming the main limitation to the clinical application of NGF (Giuliani et al., 2020).

Besides its role in angiogenesis (Calzà et al., 2001; Ahluwalia et al., 2017; Li X. et al., 2018) and its action on skin cells (Gostynska et al., 2020), NGF may act by improving local re-innervation, fundamental to the wound healing process (Kiya and Kubo, 2019). Our transcriptomic study on the CHF6467 molecule also points to the modulation of Akt/mTOR signaling as the main driver of NGF action (Giuliani et al., 2020). This pathway is in fact in involved in the wound healing process (Huang et al., 2015; Jere et al., 2019) and is regarded as a therapeutic target (Squarize et al., 2010).

## Biomaterial-Assisted Delivery

Biomaterial-based systems (nanomedicine, hydrogels and scaffolds) are a common strategy to ameliorate drug delivery, and enormous advances have been made over the last decades to assist tissue repair and regeneration by biomaterial loading different growth factors (GF) (Lee et al., 2011). The use of biomaterials has been proposed to support macromolecule topical application and to facilitate the body’s barriers crossing. For example, nanotherapeutics and nanomaterials improve the biodistribution of drugs throughout the brain for more effective treatments, not only via convection-enhanced delivery, but also via IN delivery (Keller et al., 2021).

But in spite of this progresses at the material side in controlling hydrophobicity/hydrophilicity, micro/nano-architectures, porosity, stiffness, and degradation rate, translation of materials to clinical applications is still limited due to difficulties such as scaling up reproducible manufacturing processes, the low stability of encapsulated proteins and their rapid inactivation by enzymes under physiological conditions.

### Hydrogels for NGF Delivery

Hydrogels are polymers with a 3D network and a hydrophilic structure with the potential to absorb up to thousands of times their dry weight in water (Hoffman, 2002). Their unique properties, including gelation time and gelation temperature, mechanical strength, degradability together with their good affinity and compatibility with biological tissues, make hydrogels versatile materials for drug delivery and scaffolding for tissue engineering applications (Naahidi et al., 2017). The use of hydrogels as carrier materials for NGF is an important strategy to protect this protein from inactivation, ensure its sustained delivery over time, and improve its regenerative effects. Conventional hydrogels may be unsuited to wrapping NGF due to a poor affinity to NGF, or to the lack of particular requirements such as a certain mechanical strength or shape at normal body temperature, and thermo-sensitive hydrogels may offer a valid alternative. These polymers are liquid at room temperature, changing into a 3D-network structure at normal body temperature, thus rapidly transforming from a solution to a viscoelastic gel making them particularly suitable for *in vivo* application.

#### Polysaccharide-Based-Hydrogels

Heparin poloxamer (HP) is a thermo-sensitive hydrogel with good affinity to NGF (Zhao et al., 2016). NGF-HP hydrogel maintains its thermosensitive nature and has a porous sponge-like structure which is ideal for carrying NGF and controlling its release. In an *in vivo* study on spinal cord injury (SCI) rat model, the NGF-HP hydrogel by *in situ* injection reduced the formation of a glial scar by inhibiting the generation of reactive astrocytes following SCI, promoting axon regeneration and inhibiting the formation of proteoglycans and collagen fibers, as well as promoting the formation of the new blood capillaries required for regeneration process. Moreover, and improvement in the locomotion performance was also observed.

Controlled delivery of multiple GFs to lesion areas is becoming an attractive strategy to achieve successful axonal regrowth following SCI. The HP hydrogel was therefore used for the delivery of both NGF and fibroblast growth factors (bFGF) (Hu et al., 2020). The release of these GFs from the hydrogel exhibited an initial rapid phase during the first week, and a slow sustained release. The GF-HP hydrogel was also used in a diabetic rat model with sciatic nerve crush injury to enhance the peripheral nerve regeneration with a single injection of GF-HP hydrogel. After 30 days, the GFs attenuated gastrocnemius muscle atrophy, and promoted the formation of myelinated axons, the proliferation of Schwann cells, and motor function recovery. However, the study lacks electrophysiology data and control experiments by single growth factor administration (Li R. et al., 2018).

Hydrogels carrying bioactive molecules can be used as cavity fillers in nerve conduits (NCs) for nerve reconstruction, in order to provide an ideal microenvironment for axonal regeneration. To promote the regeneration of a 5 mm gap in a rat facial nerve, an autologous vein was filled *in situ* with a thermosensitive Chitosan/β-glycerophosphate hydrogel loading NGF. While good functional recovery was achieved, the performance of the hydrogel was inferior to autologous nerve grafting (Cao et al., 2012). Alternatively, an electrospun conduit composed of aligned poly-L-Lactide-co-caprolactone (PCLC) nanofibers was filled with an NGF-loaded collagen/hyaluronan hydrogel (Jin et al., 2013). This NGF/PCLC/Hydrogel system enhanced neurite outgrowth from cultured dorsal root ganglia explants, compared to the plain PCLC/hydrogel. This result was not replicated *in vivo* to repair 10 mm gap in rat sciatic nerve, where no statistical difference in motor functional recovery and histomorphology were observed.

The combination of NGF with scaffolds presenting an ordered microstructure has also been employed (Singh et al., 2018). For example, an aligned open pore structure was generated inside a 3D printed conduit by directional cryogelation of a chitosan and gelatin solution, followed by physical absorption of NGF on the dried scaffold. When used in grafting a 15 mm gap in a rat sciatic nerve, these NCs showed significantly better results compared to the random scaffold, and even matched the performance of the autograft.

To treat chronically compressed nerves, a chitosan and sericin (CS-SS) scaffold cross-linked with genipin was developed for NGF delivery (Zhang et al., 2017). The round flake-like scaffolds were folded and adhered to the injured nerve after decompression in the *in vivo* rat model. The number and thickness of myelinated nerve fibers and axons increased, and atrophy and function impairment of the gastrocnemius muscle was suppressed.

Another scaffold-based strategy using hydrogels is aimed to obtain NGF concentration gradients, thus supporting axonal regeneration by adapting NGF release to the stage of the repair process. The use of such gradients *in vivo* to repair a challenging 20 mm gap in rat sciatic nerve was recently reported (Dodla and Bellamkonda, 2008). A polysulphone nerve guidance channel was filled with agarose hydrogel containing gradients of NGF and/or laminin, and nerve regeneration was evaluated in comparison with an autograft implant and an isotropic scaffold, containing a homogenous distribution of NGF and laminin. The anisotropic hydrogel with a concentration gradient in both NGF and laminin was the only one leading to an improved axonal regeneration, suggesting a synergistic effect, although the nerve autograft gave again the best results.

The ability of NGF to trigger the survival and neuronal differentiation of human adipose-derived stem cells (hADSCs) was exploited in the treatment of erectile disfunction in a rat model caused by an injury of the cavernous nerve. A biocompatible and biodegradable hydrogel composed of hyaluronic acid and polyethylene oxide was used as a delivery vehicle for both NGF and hADSCs by a single injection at the injury site. The hydrogel guaranteed a continuous release of NGF *in vitro* and led to an improved regeneration of the cavernous nerve, leading to a recovery of erectile function (Kim et al., 2013).

Other approaches have been used to exploit the biological effect of NGF without using the isolated protein itself, such as the use of NGF-overexpressing genetically modified hADSCs, which has been for example incorporated into a thermosensitive chitosan β-glycerophosphate/hydroxyethyl cellulose hydrogel to treat a spinal cord contusion in rats (Alizadeh et al., 2020).

#### Protein- and Peptide-Based-Hydrogels

The thermo-responsive hydrogel consisting of methoxy-poly (ethylene glycol)-*b*-poly(γ-ethyl-L-glutamate) (mPEG-PELG) was also successfully used to load NGF and obtain a controlled release (Liu et al., 2019). In a rat model, a 10 mm segment of sciatic nerve was dissected and removed, and the gap bridged using a chitosan conduit with the lumen filled of NGF/mPEG-PELG. The morphological, electrophysiological and functional analyses revealed that the chitosan scaffold with NGF/mPEG-PELG achieved superior regenerative outcomes compared to plain scaffolds or to a daily intramuscular injection of NGF.

Microporous hydrogels are another useful material. GelMA is a photo-crosslinking hydrogel composed of modified collagen components which retains cell adhesive peptide (arginyl-glycyl aspartic acid, RGD) as well as matrix metalloprotease peptides (MMP). The GelMA hydrogel was used to create an adaptable microporous hydrogel (AMH), facilitating the formation of a stable 3D porous scaffold (Hsu et al., 2019). The adaptable microporous scaffold has cell-penetrable pore sizes and was integrated with a propagating gradient of NGF in a NC. The GelMA hydrogel loaded with NGF (NGF-G-AMH@) was implanted into the 5 mm transected sciatic nerve in SD mice. NGF-G-AMH@ directed axon outgrowth of up to 4.7 mm in 4 days *in vivo*, with well aligned axons and functional recovery within 30 days post-surgery. A gel material composed of collagen, nanohydroxyapatite and carrageenan (Col/nHA/Carr) closely mimics natural bone composition and microstructure, and provides a sustained release of human NGF-A upon loading (Wang et al., 2009). In a rabbit model of mandible distraction osteogenesis (DO), a single injection of NGF-A in a Col/nHA/Carr gel at the end of a distraction period enhanced histological and morphometric nerve parameters. A more rapid recovery from the inferior alveolar nerve injury was observed due to a sustained release of NGF from the gel, which continued to exert its biological activity for a prolonged period. However, neurophysiological and behavioral studies are needed to test the effects of the locally applied NGF/Col/nHA/Carr gel on neurosensory functions (Wang et al., 2010).

The Col/nHA/NGF construct also accelerated bone formation in the same model. Although *in vitro* release studies were not conducted, the authors hypothesized that the hydrogel system prevents biodegradation of the NGF and guarantees a sustained release *in vivo*, which, combined with the intrinsic osteoconductive action of COL/nHA, led to an improvement in bone regeneration (Chao et al., 2016).

Nerve growth factor concentration gradients have been recently achieved using a modified 3D printer apparatus to get a continuous NGF concentration gradient in a silk fibroin/collagen hydrogel then subjected to directional freezing to finally obtain a 3D scaffold displaying both biochemical gradient and longitudinally oriented microchannels. It was demonstrated that both the NGF gradient and the oriented structure synergistically promoted nerve regeneration on a 15 mm gap in rat sciatic nerve *in vivo*, accelerating functional recovery, but these results were not compared to an autograft nerve repair (Huang et al., 2020).

Amphiphilic diblock co-polypeptide hydrogels (DCH) using poly-leucine and poly-glutamate or poly-lysine can be deformed and thinnered by stress, thus injected through small-bore cannula, after which they self-assemble into rigid gel networks that degrades in about 56 days. NGF could be loaded in DCH which mediate its sustained release *in vivo* inside the BBB of the CNS (Song et al., 2012). When injected in the basal forebrain, depots of DCH-NGF provided a more prolonged delivery of NGF compared with NGF injected in buffer, which induced and maintained the hypertrophy of local forebrain cholinergic neurons for at least 28 days. This hypertrophic reaction of neurons seems to follow a gradient effect from the depot, and being more evident close and attenuate far from the depot.

Nerve growth factor loaded in a gelatin-polyethylene glycol-tyramine hydrogel together with bFGF loaded in heparin-pluronic nanogels and PCL beads as a passive bulking agent was tested to treat stress urinal incontinence (SUI). The combined action of NGF and bFGF, which were released at different rates, led to a significant improvement in regeneration and reinnervation of the damaged smooth muscle around the urethra in a rat model of SUI (Oh et al., 2015).

Finally, NGF and BDNF with mimicking peptides were used to functionalize RADA16-1, a self-assembling peptide capable of forming nanofibrous hydrogels under certain conditions (Lu et al., 2018). The hydrogel was used to fill a chitosan NC to graft a 10 mm gap in rat sciatic nerve.

### Nanofibrous Electrospun Scaffolds for NGF Delivery

Among the more useful processing strategies to fabricate nanofibers, electrospinning is one of the best known methods (Greiner and Wendorff, 2007). Electrospun nanofibers with a defined micro/nanoarchitecture in terms of fiber size (fiber diameters range from a few hundreds of nanometers to tens of micrometers) and fiber orientation, have been used as a scaffold for a wide range of tissue engineering applications including neural, cardiovascular, bone and skin tissue engineering. Nanofibrous electrospun scaffolds offer a promising alternative to autologous grafting in peripheral nerve injuries, and have been extensively studied for neural tissue repair and regeneration (Ghane et al., 2021), due to their ability to act both as matrices for cells and as a delivery vehicle for various biomolecules such as NGF and glial cell line-derived neurotrophic factor (GDNF) (Liu et al., 2018; Bighinati et al., 2020). There are several reasons for the great interest in electrospun constructs in neural tissue engineering: ease of manufacture, production using a variety of natural and synthetic polymers, structural similarity with the extracellular matrix, and tunable morphology and mechanical properties. Of their various advantages, the ease of nanofiber functionalization is perhaps the most relevant, since biomolecules and drugs can easily be incorporated into electrospun scaffolds by means of several methods, including physical adsorption, blend electrospinning, coaxial electrospinning, and covalent immobilization. The nanometer scale of the fibers provides an extremely high surface-to-volume ratio, and contributes to improving biological functionality and biomolecule delivery (Ji et al., 2011). To tackle the problems related to the possible destabilization and denaturation of biomolecules such as growth factors when exposed to organic solvents in a traditional electrospinning process, variations in the technique, such as coaxial or emulsion electrospinning, have been employed to preserve the bioactivity of the incorporated biomolecules, thus enhancing the efficiency of incorporation, while controlling the release kinetics of the biomolecules at the same time.

A variety of natural and synthetic materials have been used to manufacture aligned structures for nerve regeneration, however only a few studies report significant results on the biomaterial-assisted delivery of NGF for *in vivo* applications.

In a detailed study recently published by Zhu et al. (2020) highly aligned poly(ε-caprolactone) (PCL) fibers with NGF gradients were developed for peripheral nerve regeneration. NGF was incorporated into the conduit following its manufacture, preventing the biomolecule from being negatively affected by the organic solvents used during the electrospinning process. *In vitro* studies demonstrated that the conduits enhanced and attracted the longitudinal neurite growth of the dorsal root ganglion (DRG) neurons toward their high-concentration gradient side. *In vivo*, the conduits directed a stronger longitudinal attraction of axons and migration of Schwann cells in 15 mm rat sciatic nerve defects. At 12 weeks, rats transplanted with the conduits showed satisfactory morphological and functional improvements in g-ratio and total number and area of myelinated nerve fibers, as well as sciatic function index, compound muscle action potentials, and muscle wet weight ratio, as compared to aligned conduits with uniform NGF distribution. mRNA-seq and RT-PCR results also revealed that Rap1, MAPK, and cell adhesion molecule signaling pathways were closely associated with axon chemotactic response and attraction. The performance of the NGF-gradient aligned conduits was similar to that of autografts, demonstrating the great potential of the proposed scaffolds in repairing peripheral nerve defects.

More commonly, NGF is incorporated homogeneously into the nanofibers by means of coaxial or emulsion electrospinning. In the study by Kuihua et al. (2014), an artificial nerve guidance conduit for nerve gap regeneration was designed and manufactured via coaxial electrospinning. Aligned core-shell nanofibers were obtained, with the shell made of a silk fibroin/poly(lactic-acid-co-caprolactone) blend [SF/P(LLACL)], and the core consisting of SF encapsulating NGF. This approach permitted stabilization of the NGF during the electrospinning process, and contributed to a controlled sustained release of NGF. A sustained release of biologically active NGF was observed, using ELISA and a PC12 cell-based bioassay, over a 60-day time period, although the number of neurons was lower than the positive control. The core-shell fibrous conduits were then used as a bridge implanted across a 15-mm defect in the sciatic nerve of rats. The outcome in terms of regenerated nerve at 12 weeks was evaluated by a combination of electrophysiological assessment, histochemistry, and electron microscopy, and the results, taken together, demonstrated that the NGF-aligned fibers promoted peripheral nerve regeneration significantly better than the same conduit without NGF, suggesting that the released NGF may effectively promote the regeneration of peripheral nerves. In an analogous study, very similar random core-shell nanofibers were prepared by coaxial electrospinning, consisting of a shell of P(LLA-CL) and a core of BSA/NGF (Liu et al., 2011), and the conduits used for sciatic nerve regeneration in rats. The functional and histological analyses revealed that the parameters related to the number and arrangement of regenerated nerve fibers, myelination, and nerve function reconstruction for the P(LLA-CL)/NGF group were similar to those obtained for the group where the autograph nerve was implanted, and were significantly better than for the group in which plain P(LLA-CL) electrospun fibers were implanted, even in the presence of an injection of NGF solution.

In the study by Zhang et al. (2015), a composite micro/nano-fibrous scaffold with core–shell structure was manufactured by coaxial electrospinning, combining synthetic polymers (polypyrrole, PPy) as a conductive polymer and poly(L-lactic acid, PLLA) with natural polymer and biomolecules (spider silk protein, Lysine and NGF). *In vitro* tests revealed that the scaffold was able maintain a stable structure for at least 4 months in buffered solution, with a degradation rate comparable to the nerve growth rate. Good biocompatibility and good cell adhesion with PC 12 cells were demonstrated. *In vivo* evaluation also showed that the composite fibrous conduit was effective at bridging a 20 mm sciatic nerve gap in adult rats within 10 months, and electrical stimulation through the conduit promoted Schwann cell migration and axonal regrowth.

In addition to coaxial electrospinning, emulsion electrospinning can be also used to incorporate biomolecules while preserving their bioactivity, a method used to load recombinant human NGF into the core of emulsion electrospun PLLA nanofibers (Xia and Lv, 2018). The resulting nanofibrous scaffold was then additionally loaded with recombinant human vascular endothelial growth factor (VEGF) on the surface to achieve a controlled dual-delivery of the biomolecules. *In vitro* studies showed a sequential release pattern of VEGF and NGF, with most of the VEFG released in the first few days, whereas the NGF loaded in the fiber core was continuously released for more than 1 month. After demonstrating that the scaffold enhanced neural differentiation of iPSC-NCSC cells *in vitro*, it was implanted into a critical-size defect in a rat sciatic nerve model. Footprint analysis, electrophysiological tests, and histological analysis revealed a significant improvement in neovascularization and nerve healing 3 months after surgery.

The potential of electrospinning to prepare an aligned fiber matrix able to influence the directionality and growth of axons in the CNS was investigated in the study by Colello et al. (2016). A composite material was prepared by electrospinning polydioxanone (PDO) in the presence of alginate beads incorporating NGF and chondroitinase ABC (ChABC). Upon implantation in a completely transected rat spinal cord, the composite matrices supplemented with NGF and (ChABC) promoted significant functional recovery. Examination of the conduits post-implantation revealed that electrospun aligned fibers induced a more robust cellular infiltration than random fibers. A vascular network was also generated in these matrices, since electrospun fibers acted as a growth substrate for endothelial cells. The presence of axons within the implanted electrospun matrix demonstrated that the aligned composite fibers containing NGF are able to provide trophic support and directional guidance cues to regenerating axons following spinal cord injury.

In a very recent and exhaustive study, emulsion electrospinning was used to develop innovative microenvironment-responsive (pH-responsive) immunoregulatory electrospun fibers to promote nerve function (Xi et al., 2020). PLLA-based scaffolds were manufactured, containing Rat-β-NGF microsol particles wrapped in the core of the fiber during the electrospinning process from a homogeneous and stable water-in-oil emulsion. IL-4 plasmid-loaded liposomes (pDNA) were then grafted onto the surface of the electrospun fiber scaffolds. The resulting biomimetic scaffold responded directly to the acidic microenvironment at focal areas, followed by triggered release of the IL-4 plasmid-loaded liposomes within a few hours to suppress the release of inflammatory cytokines and promote the neural differentiation of mesenchymal stem cells *in vitro*. A Sprague Dawley (SD) rat spinal hemisection model was used to investigate the *in vivo* performance on inflammation suppression, nerve regeneration and functional recovery. Once implanted into the rats with acute spinal cord injury, the scaffold showed sustained NGF release, achieved by the core-shell structure, and brought a significantly shifted immune subtype to down-regulate the acute inflammation response, reduce scar tissue formation, promote angiogenesis and neural differentiation at the injury site, and enhance functional recovery *in vivo*.

Overall, electrospinning-based technologies allow an extraordinary range of manufacturing opportunities for finely tuned design suitable for topical application. Moreover, several studies have also demonstrated that NGF bioactivity is not compromised by the electrospinning processing, making this technology suitable for applications in dermatology, but also neurosurgery and orthopedics.

### Nanomedicines for NGF Delivery

While biomacromolecules offer promising and possibly fundamental pharmaceutical treatments for controlling and tacking diseases, their action is hampered by severe limitations in delivery. This is due to chemical and physical instabilities, as well as difficulties in crossing physiological barriers, and to being accumulated and released over time at the correct site of action (Duskey et al., 2017; Tosi et al., 2019).

Conventional drug delivery strategies cannot address these limitations leading to the increase in the number of polymeric or lipidic nanomedicine (NMed) applications which have incredible potential for the medical field (Germain et al., 2020) to: (i) stabilization of the biomacromolecules by encapsulation within a polymeric or lipidic matrix, therefore assuring the required level of protection of biological activity, and (ii) a controlled release of pharmacologically relevant amounts of therapeutics at the site of action.

Depending on the material used, NMeds can be tuned in terms of size, shape, charge, binding capacity and hydrophobicity/hydrophilicity, and are easily scaled-up in view of future production on an industrial scale. This allows for a quality by design approach of an NMed with tunable characteristics to be compatible with (i) the drug characteristics; (ii) the required drug release profiles, and (iii) the characteristic or biological/pathological environment in order to control the pharmacokinetic half-life, biodistribution, stability, and overall therapeutic activity of the loaded macromolecule to be managed and regulated *ad hoc*.

One example, NGF is the most potent growth stimulating factor for cholinergic neurons and has been shown to prevent the degeneration of dopaminergic neurons, making it a promising candidate for the treatment of neurodegenerative diseases such as Alzheimer’s and Parkinson’s disease (Kurakhmaeva et al., 2008, 2009). Regarding NGF delivery by means of nanomedicines, several attempts have been made to improve loading and delivery across the BBB by engineering various polymers with different BBB targeting ligands. One example was the use of a poly(alkyl-cyanoacrylate) polymer coated with polysorbate 80 to promote BBB crossing (Kurakhmaeva et al., 2008, 2009). This coating promoted the adsorption of apolipoproteins onto the nanoparticle (NPs) surface, and the contact of the NPs with the brain capillary endothelial cells which promoted endocytosis and the intracellular release of the drug.

In the same study, NGF was adsorbed on the surface of polybutylcyanoacrylate (PBCA) NPs coated with polysorbate-80 (PS-80) surfactant for antiparkinsonian effects (Kurakhmaeva et al., 2008). Pre-treatment of the mice with NGF-loaded NPs coated with PS-80 15 min before MPTP (used to provoke parkinsonian syndrome) showed a considerable decrease in parkinsonian symptoms such as a 37% decrease in latero- and retropulsion and a 34% decrease in catalepsy as early as day 1 of observation when compared to control groups.

It is noteworthy that the total index of vertical and horizontal motor activity in the group receiving NGF-loaded NPs coated with PS-80 after MPTP was 1.78-fold higher compare to control, while treatment before MPTP induction was 2.86 times higher suggesting a potential protective effect. The effects of NGF-loaded NPs persisted for 7 and 21 days following a single injection of the neurotoxin proving to be one of the most promising NMed carriers by preventing the scavenging of the NGF by the cells of the reticuloendothelial system, prolonging circulation of these particles in the blood and increasing their concentration in cerebral vessels.

Similar experiments were conducted to explore the effect of NGF adsorbed on PBCA NPs coated with polysorbate-80 in Alzheimer’s disease. Acute amnesia in mice was induced by subcutaneous injection of scopolamine before training in the step-through passive avoidance reflex (PAR) test to determine effects on memory (Kurakhmaeva et al., 2009). The NGF-loaded PBCA NP formulation produced significantly increased latent periods in the passive-avoidance reflex (PAR) test, compared to the control animals who only received scopolamine. In contrast, systemic administration of the NGF in solution did not induce any significant changes in the mental or cognitive activity of the animals after induction of these changes by scopolamine pretreatment.

Nerve growth factor was also encapsulated into a chemically crosslinked albumin nanocarrier matrix (HSA) with ultrasmall particles of iron oxide surface-modified with apolipoprotein E to facilitate active transport into the brain and allow it to be used as a theranostic agent (Feczkó et al., 2019). The HSA NPs exhibited a size of 212 ± 1 nm, a polydispersity index (PDI) of 0.075 ± 0.022 and a zeta potential of -48.3 mV. The biocompatibility of these nanocarriers and the bioactivity of NGF were confirmed in rat pheochromocytoma (PC12) cells. Following modification of the particle surface with Apo E, the particles were able to cross the BBB and remained bioactive in terms of neurite outgrowth regulation.

In addition to Apo E, Apolipoprotein A-I was used to coat NGF lipoprotein (HDL)-mimicking NPs (Zhu and Dong, 2017). High-density lipoprotein (HDL)-mimicking NPs is a natural NP consisting of a lipid core coated with apolipoproteins, and a phospholipid monolayer which plays a critical role in the transport of lipids, proteins, and nucleic acids via its interaction with target receptors. The HDL-mimicking NPs successfully encapsulated NGF, resulting in a long half-life, prolonged release (10% over 72 h), *in vivo* stability, and increased physiological effects.

In another approach (Song et al., 2017), non-viral poly(lactic-co-glycolic acid) (PLGA) nanobubble (NBs) vectors, possessing unique advantages such as targeting, slow release and penetration, were used as gene carriers to deliver NGF. PLGA is one of the most successful polymers used in the development of drug delivery systems, offering excellent biocompatibility and biodegradability of NPs (Tan et al., 2013; Ruozi et al., 2015).

The NGF/PLGA NBs formed by double emulsion was 215.3 ± 55.29 nm, the PDI was 0.027 and the zeta potential was –11.3 ± 5.65 mV. It underwent Ultrasound (US)-mediated destruction to deliver NGF, resulting in diminished histological injury, neuron loss and neuronal apoptosis, and increased BBB scores in a rat model of spinal cord injury.

Chitosan, another widely used biodegradable and biocompatible polymer was used by Razavi et al. (2019) to encapsulate NGF in chitosan nanoparticles (NGF-CNPs). NGF-CNPs were characterized by photon-correlation spectroscopy analysis, which showed a mean NGF-CSNP diameter of 147.04 ± 8.09 nm, and a good stability of the nanoparticle surface charge (36.47 ± 1.88 mV). The encapsulation efficiency of NGF in chitosan nanoparticles is 83.93 ± 2.45%. These NMeds were evaluated for their differentiation potential of human adipose-derived stem cells (h-ADSCs) to Schwann-like cells as a source for treating various diseases such as peripheral nerve regeneration multiple sclerosis and diabetic neuropathy (Razavi et al., 2019). NGF-CNPs demonstrated no cytotoxicity and offered a sustained release of NGF reaching 74.63 ± 2.07% over 7 days without any initial burst release leading to an increased differentiation of h-ADSCs into Schwann-like cells and myelinating capacity *in vitro*.

Similarly, NGF was encapsulated into NPs [n(NGF)] of methacryloyloxyethyl phosphorylcholine (MPC), analogous to choline and acetylcholine, and polylactic acid (PLA) diacrylate to provide proof of CNS targeting in healthy mice following intravenous injection (Xu et al., 2019). The MPC-PLA exhibited an average diameter of 30.3 ± 3.6 nm under TEM, and a zeta potential of 24 mV. PC12 cells were treated with native NGF and NGF NPs to assess the activity of the NGF released from the nanocapsules. When the NGF was released from the NPs, it induced the differentiation and neurite outgrowth of these cells through intracellular pathways. The therapeutic benefit of n(NGF) for CNS repair following injury was evaluated in a mouse model of compression-induced acute spinal cord injury. After 21 days, extensive ankle movements and occasional plantar stepping was observed, representing a significant functional recovery in locomotion.

Besides these results, widely reviewed in the past literature (Ruozi et al., 2012; Srikanth and Kessler, 2012; Angelova et al., 2013; Géral et al., 2013), we would like to highlight some key factors which may significantly improve the chances of success for NMed in the field of biomacromolecule delivery.

Regarding the choice of NMed, its design and production, a major concern, still hotly debated, regards two main aspects of nanoproduction. The first is the absolute conviction that it is possible to develop one single nanomedicine for every drug (or macromolecule), the so-called “magic bullet” (Strebhardt and Ullrich, 2008; Flühmann et al., 2019), is neither more a reality nor the future. This erroneous view of the *magic bullet* led to many years of research without any real or concrete advances in the translatability of NMeds to a clinical setting. Therefore, it is pivotal, when approaching a NMed design to consider the future NMed as a single product together with the embedded drug.

The second aspect, especially when considering biomacromolecules such as NGF, proteins or enzymes, relates to the stability of the biological drugs throughout the preparation procedure and during storage. The greatest drawback concerns the requirements of nanoproduction (such as stirring, heating, sonication, organic solvents, etc.), which severely impact the stability and maintenance of the biological drug’s pharmacological activity (Duskey et al., 2020). Some of these requirements relate to the polymer/lipid used in the formulation, and should be carefully designed and always adapted to the “stability features” of the embedded drug. Failure to take these aspects into consideration when designing the NMed risks rendering the loaded drug ineffective, thus defeating its purpose.

## Gene Therapy and Cell-Assisted Biodelivery

Nerve growth factor cell and gene therapy for the CNS, in particular to target cholinergic degeneration in AD, has been investigated in preclinical models and also tested in human studies (Hosseini et al., 2018; Mitra et al., 2019), particularly in the United States (Rafii et al., 2018) and Sweden (Eyjolfsdottir et al., 2016).

Results from the US study on gene therapy in AD patients have recently been reviewed to include CNS analysis following autopsy. Intraparenchymal adeno-associated virus serotype 2 (AAV2)-NGF delivery was safe but did not improve cognition. Neuropathological analysis then aimed to establish whether (AAV2)-NGF engaged the target cholinergic neurons of the basal forebrain. Patients with clinically diagnosed early- to middle-stage AD received a total dose of 2 × 1011 vector genomes of AAV2-NGF by stereotactic injection of the nucleus basalis of Meynert. Following a mean survival of 4.0 years, AAV2-NGF targeting, spread, and expression indicated that NGF gene expression persisted for at least 7 years at the sites of AAV2-NGF injection. However, the mean distance of AAV2-NGF spread was only 0.96 ± 0.34 mm, indicating that NGF did not directly reach the cholinergic neurons at any of the 15 injection sites. Given that AAV2-NGF did not directly engage the target cholinergic neurons, the authors cannot conclude that growth factor gene therapy is effective for AD (Castle et al., 2020).

In the Swedish study, biodelivery of NGF (NGF-ECB) by encapsulated cell was used in AD patients in a first-in-human study. Results were gathered from a third dose cohort of patients with mild to moderate AD, receiving second-generation NGF-ECB implants with improved NGF secretion, in an open-label, phase Ib dose escalation study with a 6-month duration. Each patient underwent stereotactic implant surgery with four NGF-ECB implants generated using the Sleeping Beauty transposon gene expression technology targeted at the cholinergic basal forebrain, resulting in production of about 10 ng NGF/device/day. The data derived from this patient cohort demonstrate the safety and tolerability of sustained NGF release by a second-generation NGF-ECB implant to the basal forebrain. Moreover, the patients’ responses to the NGF-treatment indicated that approximately half of the patients responded to the ECB-NGF-treatment with increased cholinergic markers (e.g., ChAT activity) in the CSF, correlating to improved cognition and brain glucose metabolism (Karami et al., 2015), less brain atrophy (Ferreira et al., 2015), and normalization of the EEG-pattern (unpublished data).

## Discussion

The discovery of endogenous GF production of GFs during adulthood as well as during development opened new perspectives for mature CNS biology, moving away from the dogma of prominent histologist Ramon y Cajal: “Once development ended, the founts of growth and regeneration of the axons and dendrites dried up irrevocably. In the adult centers, the nerve paths are something fixed, ended, and immutable. Everything may die, nothing may be regenerated. It is for the science of the future to change, if possible, this harsh decree.” We are now in the science of the future, knowing that endogenous regeneration may occur in the mature CNS, albeit to a limited extent. NGF can now be produced using human recombinant technologies, and molecules which can limit adverse side-effects are available either as modified full-length proteins or as TrkA short peptides analogs. However, we still need to better understand that NGF-based therapies should be considered as “hormonal” therapies rather than conventional pharmacological therapies, in view of the endogenous production of NGF. We also need to protect the molecule from protein degradation, and promote the crossing of blood-tissue barriers, in order to bring the appropriate molecule concentration to the appropriate place for the appropriate time. The use of modern biomaterial technologies is an essential strategy for rapidly achieving this goal. Scaffolds obtained by different fabrication procedures, such as hydrogels and composite materials are providing significant indications about efficacy of NGF delivery in peripheral nerve, but also in other tissues repairs, as bone, while nanoparticle conjugation is regarded as a promising strategy also to overcome physiological barriers.

However, in view of clinical translation, we also need to move forward in preclinical research, which, at the present, provides a puzzling and incomplete picture of biomaterial potentiality. In particular, we need well-designed proof-of-concept studies for both safety and efficacy, thus including appropriate control groups and defined functional end-points in the efficacy studies, and the Good Laboratory Practice standard for safety studies. Because of this, a more stringent interdisciplinary collaboration would be desirable, such as a more stringent editorial policy in both biomaterial and biomedical journals.

## Author Contributions

All authors listed have made a substantial, direct and intellectual contribution to the work, and approved it for publication.

## Conflict of Interest

The authors declare that the research was conducted in the absence of any commercial or financial relationships that could be construed as a potential conflict of interest.
